# Pesticides and Parkinson’s Disease

**DOI:** 10.1100/tsw.2001.35

**Published:** 2001-05-01

**Authors:** Todd B. Sherer, Ranjita Betarbet, J. Timothy Greenamyre

**Affiliations:** Department of Neurology, Emory University, Atlanta, GA 30322, USA

**Keywords:** neurodegeneration, mitochondria, rotenone, complex I

## Abstract

Parkinson’s disease (PD), a common neurodegenerative disorder affects approximately 1% of the population over 65. PD is a late-onset progressive motor disease characterized by tremor, rigidity (stiffness), and bradykinesia (slowness of movement). The hallmark of PD is the selective death of dopamine-containing neurons in the substantia nigra pars compacta which send their projections to the striatum and the presence of cytoplasmic aggregates called Lewy bodies [1-2]. Most cases of PD are sporadic but rare cases are familial, with earlier onset. The underlying mechanisms and causes of PD still remain unclear.

Recently, a paper by Betarbet et al., published in the December issue of *Nature Neuroscience*, implicates mitochondrial dysfunction and pesticide exposure as possible causes for PD. This study showed that rats, chronically infused with rotenone, showed features similar to those observed in PD patients including degeneration of the dopaminergic nigrostriatal pathway and the presence of cytoplasmic inclusions reminiscent of Lewy bodies [3]. Rotenone, a commonly used pesticide, is also a potent inhibitor of mitochondrial function. This novel model of PD illustrates that environmental toxins and mitochondrial dysfunction may play an important role in the pathogenesis of PD.

Many hypotheses exist to explain the etiology of PD. Genetic analysis of families with a high incidence of PD has uncovered a number of mutations including mutations in genes encoding parkin and ubiquitin carboxy-terminal hydrolase-L1 that lead to diseases somewhat distinct from idiopathic cases of PD [4-6]. In contrast, mutations found in the gene for a-synuclein, a protein that was determined to be a major component of Lewy bodies [7], suggested that a-synuclein aggregation and Lewy body formation may be an important trigger in the disease process [8-9]. Additionally, epidemiological studies suggest an association between exposure to environmental toxins and PD. Individual case studies have linked PD with exposure to the herbicide, paraquat, and the fungicide maneb [10]. Exposure to paraquat and maneb induce nigrostriatal dopaminergic degeneration in animal models [11-12]. Furthermore, in the 1970s, a group of intraveneous heroin addicts accidentally ingested MPTP and quickly developed features of PD [13]. The active metabolite of MPTP, $MPP^+$, was found to be a mitochondrial toxin [14]. Specifically, $MPP^+$ inhibited complex I of the electron transport chain responsible for cellular energy production. This result lead the discovery that PD patients express systemic complex I defects in a number of tissues including muscle and brain [15-17]. The recent study by Betarbet et al. tested a model of PD based upon chronic systemic administration of rotenone. This model simultaneously addresses the role of environmental toxins, mitochondrial dysfunction, and a-synuclein in PD pathogenesis.

In the rotenone model of PD, rats were continuously infused with low doses of rotenone through jugular vein cannulation for 1 to 5 weeks. As a pesticide and mitochondrial toxin, the use of rotenone offered the opportunity to link the role of environmental agents and mitochondrial dysfunction in PD. Since rotenone is lipophilic and is able to cross biological membranes, mitochondrial function (complex I inhibition) was uniformly reduced throughout the brain. This is in contrast to $MPP^+$, that solely inhibits complex I activity in dopamine-containing cells. Systemic rotenone administration caused highly selective nigrostriatal dopaminergic degeneration that was associated behaviorally with decreased activity and in some cases rigidity. The GABAergic and cholinergic pathways of the striatum were not affected. Nigral neurons in rotenone-treated rats accumulated cytoplasmic inclusions containing a-synuclein. These results indicate that chronic exposure to rotenone, a common pesticide and potent mitochondrial toxin, reproduced the anatomical, neurochemical, behavioral, and neuropathological features of PD [3]. These results also suggest that the dopamine-containing cells of the substantia nigra do, in fact, have an intrinsic sensitivity to complex I inhibition.

Chronic exposure to rotenone, a mitochondrial toxin and commonly used pesticide, mimicked characteristic features of PD. These results propose systemic mitochondrial inhibition as well as environmental agents as potential causal factors for PD. However, the mechanisms through which rotenone causes selective nigrostriatal dopaminergic degeneration and the formation of a-synuclein positive cytoplasmic aggregates are unknown. The development of this novel model of PD has opened new avenues for the study of PD pathogenesis, which, in turn, should yield innovative therapeutic targets.

| | | |
|---|---|---|
| Received: | March | 5, 2001 |
| Accepted: | March | 26, 2001 |
| Published: | May | 1, 2001 |
